# Breed identification using breed-informative SNPs and machine learning based on whole genome sequence data and SNP chip data

**DOI:** 10.1186/s40104-023-00880-x

**Published:** 2023-06-01

**Authors:** Changheng Zhao, Dan Wang, Jun Teng, Cheng Yang, Xinyi Zhang, Xianming Wei, Qin Zhang

**Affiliations:** Shandong Provincial Key Laboratory of Animal Biotechnology and Disease Control and Prevention, College of Animal Science and Veterinary Medicine, Shandong Agricultural University, Tai’an, 271018 China

**Keywords:** Breed identification, Breed-informative SNPs, Genomic breed composition, Machine learning, Whole genome sequence data

## Abstract

**Background:**

Breed identification is useful in a variety of biological contexts. Breed identification usually involves two stages, i.e., detection of breed-informative SNPs and breed assignment. For both stages, there are several methods proposed. However, what is the optimal combination of these methods remain unclear. In this study, using the whole genome sequence data available for 13 cattle breeds from Run 8 of the 1,000 Bull Genomes Project, we compared the combinations of three methods (Delta, *F*_ST_, and *I*_n_) for breed-informative SNP detection and five machine learning methods (KNN, SVM, RF, NB, and ANN) for breed assignment with respect to different reference population sizes and difference numbers of most breed-informative SNPs. In addition, we evaluated the accuracy of breed identification using SNP chip data of different densities.

**Results:**

We found that all combinations performed quite well with identification accuracies over 95% in all scenarios. However, there was no combination which performed the best and robust across all scenarios. We proposed to integrate the three breed-informative detection methods, named DFI, and integrate the three machine learning methods, KNN, SVM, and RF, named KSR. We found that the combination of these two integrated methods outperformed the other combinations with accuracies over 99% in most cases and was very robust in all scenarios. The accuracies from using SNP chip data were only slightly lower than that from using sequence data in most cases.

**Conclusions:**

The current study showed that the combination of DFI and KSR was the optimal strategy. Using sequence data resulted in higher accuracies than using chip data in most cases. However, the differences were generally small. In view of the cost of genotyping, using chip data is also a good option for breed identification.

**Supplementary Information:**

The online version contains supplementary material available at 10.1186/s40104-023-00880-x.

## Background

Breed identification can have several practical applications including (a) the management of livestock genetic resources [1], (b) understanding and evaluating the breeding history and breed purity of a certain animal breed [2, 3], (c) implementation of breeding strategies and plans [4], (d) inference of product provenance to improve supply chain integrity [5–7], and (e) conservation of local-specific species [2, 8]. The general principle that makes it possible to allocate animals to specific breeds relies on the genetic heterogeneity present amongst breeds that might be higher than within breeds [3]. SNPs are increasingly popular as breed identification markers because they are highly abundant and widespread in the genome. Genome-wide SNP markers can be discovered and genotyped by using a SNP array or genome sequencing [9, 10]. Many commercial SNP chips have been used to capture breed-informative markers useful for several applications [7, 11–13]. However, there are few studies on breed identification based on whole genome sequencing data.

Breed identification usually involves a two-stage approach, namely (a) detection of breed-informative SNPs based on a reference population consisting of multiple known breeds and (b) assignment of individuals of unknown breed to their corresponding breeds based on the breed-informative SNPs [14–16]. Several statistical methods have been proposed to obtain highly breed-informative SNPs among the genome-wide abundant markers, such as Delta [17], which has been used in human [18] and pigs [19], pairwise Wright’s *F*_ST_ [20], which has been extensively applied to identify breed-informative SNPs, population structures, and selection signature in livestock [21–23], and informativeness for assignment (*I*_n_) [24], which takes into account self-reported ancestry information from sampled individuals and has been used in the inference of ancestry [24, 25]. Besides, there were some studies that highlighted the impact of minor allele frequency (MAF) and linkage disequilibrium (LD) on the selection of breed-informative SNPs [11, 26].

Based on the detected breed-informative SNPs, assignment of individuals to their breeds is conducted through a classification procedure. With the advent of artificial intelligence, some machine learning methods have been used in this stage [7, 16, 27], such as Artificial Neural Network (ANN), Random Forest (RF) [7, 28], Naïve Bayes (NB) [12], Support Vector Machine (SVM) [29], and K-Nearest Neighbor (KNN) [12]. However, there were few investigations on the combination of different detection methods of breed-informative SNPs and different machine learning methods and the optimal combination between these methods remains unclear.

Alternatively, breed identification can also be attained by estimating genomic breed composition (GBC). In this context, a linear regression model is used to estimate the GBC of individuals to be identified, where their SNP genotypes are regressed to the allele frequencies of different breeds in the reference population. The GBC for a breed is estimated as the ratio of that breed’s regression coefficient over the sum of all regression coefficients [15, 16, 30, 31]. The GBC of an individual for a breed also represents the probability that the individual belongs to this breed. An advantage of the GBC analysis is that it can be used to estimate whether an individual is a purebred animal of a given breed (if the corresponding probability is equal to or close to one, say > 0.9) or a crossbred animal with estimated GBC of involved breeds. This is particularly useful for estimating heterosis and breed additive effects which facilitates cross breed genetic evaluation allowing the comparison to selection candidates across breed. This is also important for monitoring the quality and genuineness of animal products.

In this study, using the whole genome sequence data available for 13 cattle breeds from Run 8 of the 1,000 Bull Genomes Project, we evaluated the accuracies of different combinations of three methods for breed-informative SNPs detection and five machine learning methods for breed assignment. In addition, we proposed to integrate the different methods for breed-informative SNPs detection and different machine learning methods. The effects of reference population size and number of most breed-informative SNPs were investigated. Meanwhile, we evaluated the identification accuracy using SNP chip data. We also performed GBC analysis to evaluate the purity of these breeds.

## Materials and methods

### Animals and genotypes

We accessed the database from Run 8 of the 1000 Bull Genomes Project [32]. The original database contains sequence data of 4,109 bulls with genotypes of 64,644,013 SNPs. From this resource, we selected bulls from breeds with more than 30 bulls. All bulls with a sequencing depth of at least 10× were selected from each of these breeds. We obtained SNP data of 1095 bulls of 13 breeds. Table 1 shows the number of animals and sequencing depths of the 13 breeds. Quality control of the SNP data was carried out using PLINK 1.9 [33]. SNPs were filtered out if the following requirements were not attained: (i) being biallelic, (ii) 100% genotyping rate (several methods used in this study for detection of informative SNPs or classification do not allow any missing values), or (iii) locating on autosomes. Finally, a total of 60,062,797 SNPs was used in this study.

**Table 1:** Numbers of bulls and sequencing depths of the 13 breeds

Breed	Code	*N*	Sequencing depth		
			**Min**	**Max**	**Mean (SD)**
Normande	NMD	31	10.03	30.76	12.68 (4.59)
Yakut	YKT	31	10.11	20.97	13.45 (2.93)
Gelbvieh	GEL	33	10.03	21.13	14.83 (3.67)
Limousin	LIM	39	10.03	20.88	12.73 (3.00)
Montbeliarde	MBL	55	10.18	27.73	13.28 (4.30)
Hereford	HF	67	10.13	45.48	15.22 (5.48)
Norwegian Red	NWR	78	10.01	33.42	11.10 (2.62)
Charolais	CHA	72	10.02	21.35	13.48 (3.25)
Simmental	SIM	83	10.01	33.90	15.48 (5.21)
Brown Swiss	BS	120	10.12	60.01	18.34 (8.84)
Jersey	JER	127	10.05	31.55	14.12 (3.76)
Angus	ANG	159	10.03	36.91	17.78 (5.36)
Holstein	HOL	200	10.00	59.09	17.43 (8.75)

The 1,095 bulls were divided into a reference population and a test population. The reference population contained the top 30 bulls of each breed with respect to their sequencing depth (390 in total), which was used to detect breed-informative SNPs and to train the classification model. The test population contained the remaining bulls of each breed (705 in total), which was used to evaluate the performance of different methods for breed identification.

For each of the 1,095 bulls, we also generated its SNP chip data corresponding to the widely used 5 types of cattle SNP chips, including Illumina Bovine SNP50 BeadChip (50K), GGP Bovine HD (80K), GGP Bovine 100K (100K), GGP Bovine HDv3 (150K), and Illumina Bovine HD BeadChip (777K). To maintain consistency with the sequencing data, we first mapped the chip SNPs to the bovine reference genome ARS-UCD1.2 [34], and then extracted SNPs from the original database with 64,644,013 SNP genotypes according to their genome position.

### Methods for detection of breed-informative SNPs

Firstly, genotype quality control was carried out with PLINK1.9 [33], and SNPs with MAF less than 0.05 or with linkage disequilibrium (LD) *r* ^2^ > 0.2 within a 50-SNPs-window were excluded, resulting in 789,141 SNPs.

Secondly, we used three methods to detect breed-informative SNPs by using the reference population, i.e., Delta [17], pairwise Wright’s *F*_ST_ [20], and informativeness for assignment [24].

#### Delta

The informative score of a SNP is measured with the Delta value, which defined as follows. For any two breeds *i* and *j*, calculate

$$\delta =|{p}_{A}^{i}-{p}_{A}^{j}|$$

where ${p}_{A}^{i}$ and ${p}_{A}^{j}$ are frequencies of allele A in breeds *i* and *j*, respectively. This ${\delta }_{ij}$ value is calculated for all pairwise combinations of all breeds, and then the final Delta value is the average value of all pair-wise $\delta$ values.

#### Pairwise Wright’s *F*_ST_

Pairwise Wright’s *F*_ST_ is computed in the same way as that for Delta. For any two breeds *i* and *j*, calculate

$${F}_{ST}=\frac{{H}_{T}-{H}_{S}}{{H}_{T}}$$

where ${H}_{T}=2{p}_{A}{p}_{B}$ is the expected heterozygosity in the two breeds together, ${H}_{S}={p}_{A}^{i}{p}_{B}^{i}+{p}_{A}^{j}{p}_{B}^{j}$ is the average expected heterozygosity of the two breeds. Here, ${p}_{A}$ is the frequency of allele A in the two breeds, ${p}_{A}^{i}$ and ${p}_{A}^{j}$ are frequencies of allele A in breed *i* and *j*, respectively. Notations for subscript B are defined similarly. Then, all pairwise ${F}_{ST}$ values are averaged to get the final ${F}_{ST}$ value.

#### Informativeness for assignment (*I*_n_)

The informative score of a SNP is measured with the *In* value as follows:

$${\mathrm{I}}_n=\sum_{j=1}^N\left(-p_j{\text{log}}_2p_j+\sum_{i=1}^K\left(p_{ij}{\text{log}}_2p_{ij}\right)/K\right)$$

where *N* is number of SNPs, *K* is number of breeds, ${p}_{ij}$ is the frequency of SNP *j* in breed *i*, and ${p}_{j}$ is the average frequency of SNP *j* across the *K* breeds. It is defined that 0 log_2_0 = 0.

For each method, the informative scores for all SNPs were calculated and ranked. The top *M* SNPs were taken as most breed-informative (MBI) SNPs. To explore the effect of number of MBI SNPs on the accuracy of breed identification, different numbers of MBI SNPs (*M* = 200, 500, 1,000, 1,500, 2,000) were considered and compared.

The software TRES [18], in which the above three methods are implemented, was used to obtain the breed-informative SNPs and the lists of ranked SNPs.

In addition, we also tried to integrate the three methods by taking the common SNPs of MBI SNPs revealed by the three methods and then regarded these common SNPs as the MBI SNPs. We called this method DFI.

### Classification methods for breed assignment

The MBI SNPs revealed from the reference population were used to train the machine learning models through alignment of the SNPs of individuals in the test population with the MBI SNPs of the individuals in the reference population. Five machine learning methods were considered: Naive Bayes, Support Vector Machine, K-Nearest Neighbor, Random Forest, and Artificial Neural Network.

NB is a kind of simple probabilistic classification methods based on Bayes' theorem with the assumption of independence between features [35]. The naiveBayes function of the R package e1071 (https://cran.r-project.org/web/packages/e1071/) was used to perform NB classification.

SVM applies a data transformation that project the data into a higher dimensional space to find a separating decision surface, which is a boundary that maximally separates classes [36]. The svm function of R package e1071 (https://cran.r-project.org/web/packages/e1071/) was used to perform SVM classification.

KNN conducts classification tasks by first calculating the distance between the test sample and all training samples to obtain its nearest neighbors and then assigning the test samples with labels by the majority rule on the labels of selected nearest neighbors [37]. The knn function of R package class (https://cran.r-project.org/web/packages/class/) was used to perform KNN classification.

RF makes use of decision trees and builds a forest of decision trees, each tree is based on a different subset of features and observations of the data [38]. The randomForest function of R package randomForest (https://cran.r-project.org/web/packages/randomForest/) was used to perform RF classification.

ANN is inspired by the structure and behavior of biological neural networks and consists of a set of source nodes that constitute the input layer, one or more hidden layers of computation nodes and an output layer [39]. The nnet function of R package nnet (https://cran.r-project.org/web/packages/nnet/) was used to perform ANN classification.

The detailed parameters setting for these machine learning classification methods were shown in Additional file 1: Table S1.

### Breed identification with different types of SNP data

Three types of SNP data were considered for breed identification of the test individuals, i.e., (1) both reference and test populations were genotyped by sequencing, (2) both reference and test populations were genotyped by generated SNP chip (50K, 80K, 100K, 150K, and 777K), and (3) the reference population was genotyped by sequencing, while the test population was genotyped by generated SNP chip. In this case, the chip genotype data of the test individuals were imputed to sequence data using Beagle v5.1 [40]. The sequence data of 2078 bulls of the above 13 breeds obtained from Run 8 of the 1000 Bull Genomes Project [32] was used as a reference panel. The imputation accuracy was measured with Pearson correlation coefficient between imputed genotypes and typed genotypes [41].

### Evaluation of different breed identification pipelines

The test population with 705 individuals was used to evaluate the performance of different identification pipelines (i.e., combinations of different breed-informative detection methods and different machine-learning classification methods). Each machine-learning classification was repeated 50 times. The performance of breed identification was evaluated by accuracy defined as follows:

$$Accuracy=\frac{1}{50}\sum_{i=1}^{50}\frac{{N}_{T}}{{N}_{T}+{N}_{F}}$$

where ${N}_{T}$ is number of individuals which were correctly assigned to their breeds of origin and ${N}_{F}$ is the number of individuals which were wrongly assigned.

To test the effect of reference population size on the accuracy of breed identification, in addition to the size with 30 individuals per breed, we also considered sizes with 10 and 20 individuals per breed. These individuals were randomly sampled from the 30 individuals and three repeated sampling were performed.

### Estimation of genomic breed composition

The GBC of the animals in the test population were estimated using all of the 789,141 SNPs based on the following linear regression model:

$$\bf {\varvec{y}}=1\mu +{\varvec{X}}{\varvec{b}}+{\varvec{e}}$$

where ***y*** is the vector of genotypes for a given test animal for all SNPs, **1** is an unit vector, *μ* is the overall mean, ***X*** is a matrix containing the allele frequencies of each SNP in each of the 13 breeds in the reference population, ***b*** is a vector of regression coefficients for the 13 breeds, and ***e*** is a vector of random residuals with distribution of $N(0, {\varvec{I}}{\sigma }_{e}^{2})$ with ${\sigma }_{e}^{2}$ being the residual variance and ***I*** being an identity matrix. The GBC of a given animal for a breed is defined as the ratio of the corresponding regression coefficient to the sum of regression coefficients for all of the 13 breeds.

## Results

### Both reference and test populations genotyped by sequencing

#### Detection of breed-informative SNPs

**Fig. 1:**
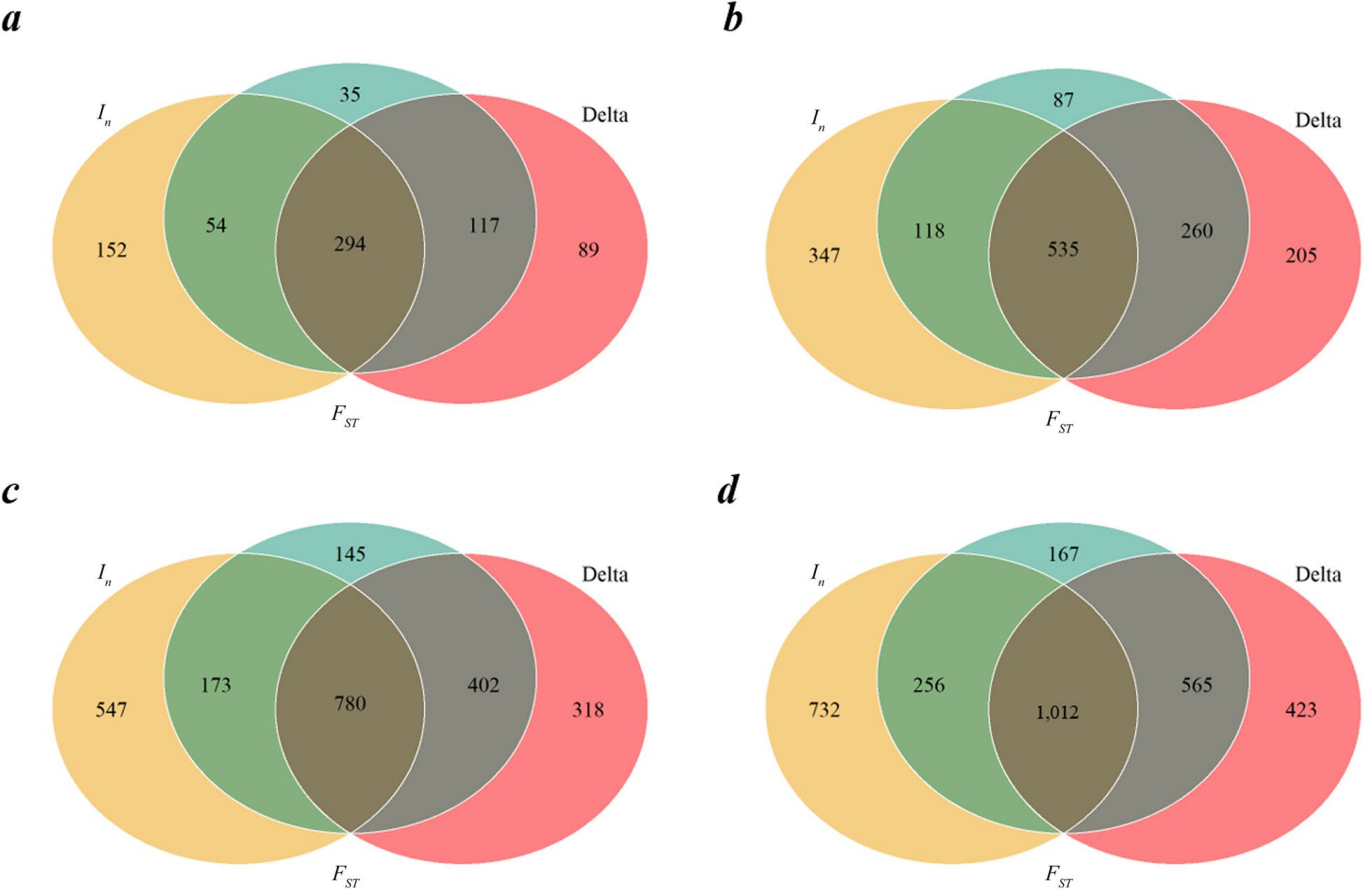
Overlaps of the most breed-informative SNPs revealed by Delta, *F*_ST,_ and *I*_n_ with the reference population size of 30 individuals per breed. **a**, **b**, **c** and **d** refer to the most breed-informative SNPs were 500, 1,000, 1,500, and 2,000, respectively

The three breed-informative SNPs detecting methods (Delta, *F*_ST_ and *I*_n_) were compared using the reference population with 30 bulls per breed. Figure 1 shows that the MBI SNPs detected by the three methods were not consistent. For the given numbers of MBI SNPs, 500, 1,000, 1,500, and 2,000, the percentages of common SNPs among the MBI SNPs revealed by the three methods were 58.80%, 53.50%, 52.00% and 50.60%, respectively. The *F*_ST_ method was most consistent with the other two methods with over 90% SNPs overlapping with that detected by Delta or *I*_n_, while the *I*_n_ method was most inconsistent with less than 70% SNPs overlapping with that detected by *F*_ST_ or Delta. The common SNPs between *I*_n_ and Delta were the same as those among the three methods.

#### Accuracies of breed identification

**Fig. 2:**
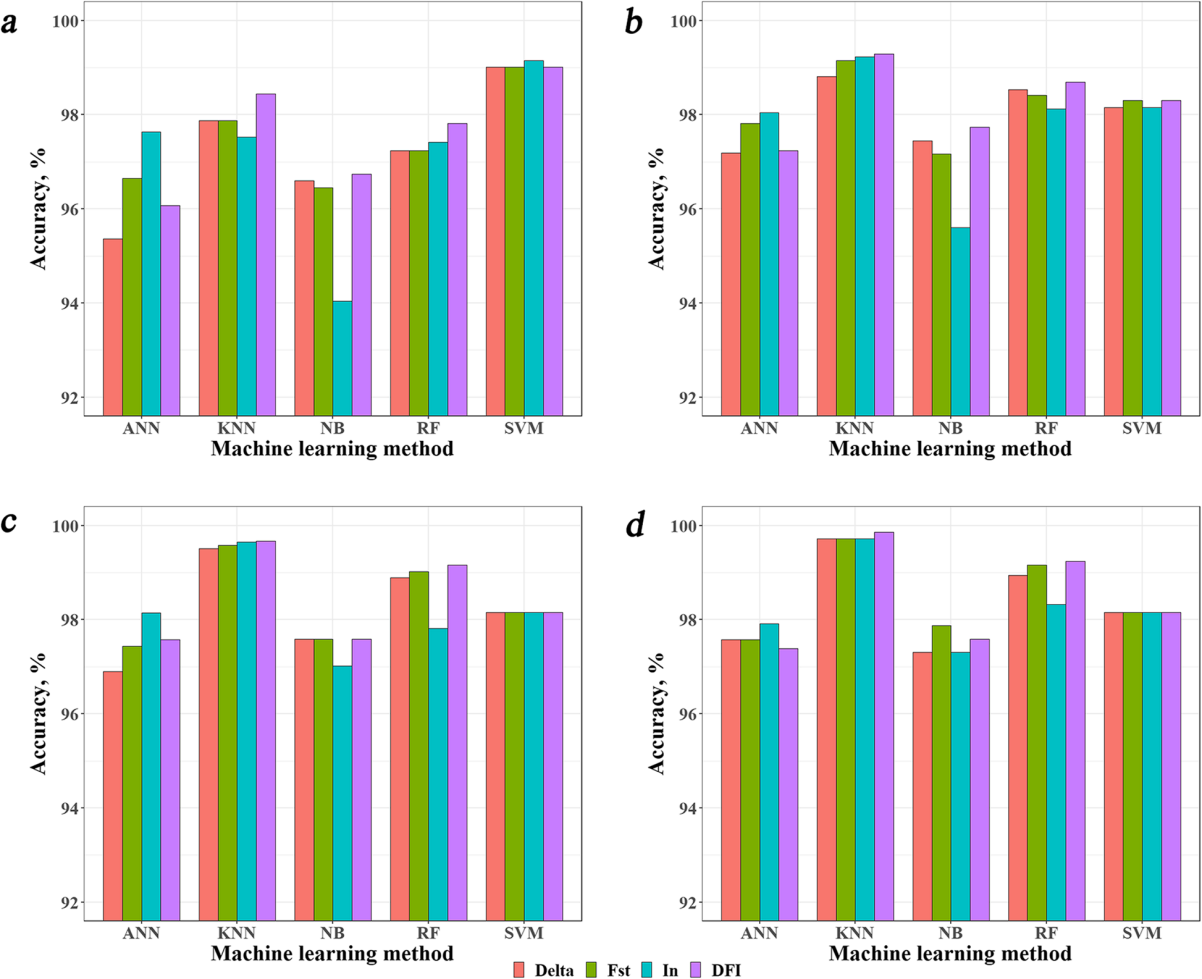
Identification accuracies of different combinations of breed-informative SNPs detection methods (Delta, *F*_ST_, *I*_n_, and DFI) and machine learning classification methods (ANN, KNN, NB, RF and SVM) with the reference population size of 30 individuals per breed. **a**, **b**, **c** and **d** refer to the most breed-informative SNPs were 500, 1,000, 1,500, and 2,000, respectively. *ANN* Artificial Neural Network, *KNN* K-Nearest Neighbor, *NB* Naive Bayes, *RF* Random Forest, *SVM* Support Vector Machine

We first compared the accuracies of breed identification of different pipelines when the reference population size was 30 bulls per breed (Fig. 2 and Additional file 1: Table S2). The results showed that when the number of MBI SNPs was 1,000, 1,500, and 2,000, the KNN-based pipelines performed better than all other pipelines (accuracies reached over 99%), followed by the RF-based pipelines (accuracies reached over 98%), while when the number of MBI SNPs was 500, the SVM-based pipelines performed the best (accuracies also reached 99%), followed by the KNN-based pipelines. The NB-based and the ANN-based pipelines performed the worst in general. When comparing the different breed-informative SNP detection methods within a machine-learning method, in general, the DFI method performed better than or equally well as the other methods, although the differences were small, except for the ANN-based pipeline, where the *I*_n_ method performed the best. It should be noted that for the DFI method, the number of MBI SNPs referred to the number of common MBI SNPs revealed by the Delta, *F*_ST_, and *I*_n_ methods. For example, the 1,000 MBI SNPs for DFI came from three sets of around 2,000 MBI SNPs revealed by Delta, *F*_ST_, and *I*_n_. Generally, the accuracies increased with the increase of number of MBI SNPs, except for the SVM-based pipelines which performed the best when the number of MBI SNPs was 500.

Table 2 shows the detailed incorrectness for each breed based on 2,000 MBI SNPs of DFI. It can be seen that for ANN incorrect assignment occurred almost in all breeds with an overall error rate of 2.61%, while for KNN only one out of 705 individuals was incorrectly assigned. It should be noted that the majority of the incorrect assignments happened in Brown Swiss.

**Table 2:** Numbers of incorrect assignment (Mean (SE) over 50 replications) in different breeds by different machine learning methods with reference population size of 30 individuals per breed and 2,000 most breed-informative SNPs revealed by DFI

Breed	No anim	Machine learning				
		**ANN**	**KNN**	**NB**	**RF**	**SVM**
NMD	1	0.10 (0.10)	0.00	0.00	0.00	0.00
YKT	1	0.00	0.00	0.00	0.00	0.00
GEL	3	0.20 (0.05)	0.00	0.00	0.00	0.00
LIM	9	0.30 (0.07)	0.00	0.00	0.00	0.00
MBL	25	0.30 (0.13)	0.00	0.00	0.00	0.00
HF	37	0.40 (0.17)	0.00	0.00	0.00	0.00
NWR	48	1.40 (0.16)	0.00	0.00	0.00	0.00
CHA	42	1.80 (0.23)	0.00	0.00	0.04 (0.03)	0.00
SIM	53	3.60 (0.44)	1.00 (0.00)	1.00 (0.00)	0.06 (0.03)	0.00
BS	90	6.20 (0.30)	0.00	13.00 (0.00)	4.00 (0.17)	12.00 (0.00)
JER	97	0.20 (0.06)	0.00	0.00	0.00	0.00
ANG	129	0.80 (0.17)	0.00	2.00 (0.00)	1.02 (0.02)	1.00 (0.00)
HOL	170	3.10 (0.32)	0.00	1.00 (0.00)	0.24 (0.06)	0.00
Total	705	18.40 (0.49)	1.00 (0.00)	17.00 (0.00)	5.36 (0.18)	13.00 (0.00)

*ANN* Artificial Neural Network, *KNN* K-Nearest Neighbor, *NB* Naive Bayes, *RF* Random Forest, *SVM* Support Vector Machine

Since the ANN and NB based pipelines performed worse than the other pipelines, we discarded these pipelines in the subsequent analysis.

#### Effect of reference population size

**Fig. 3:**
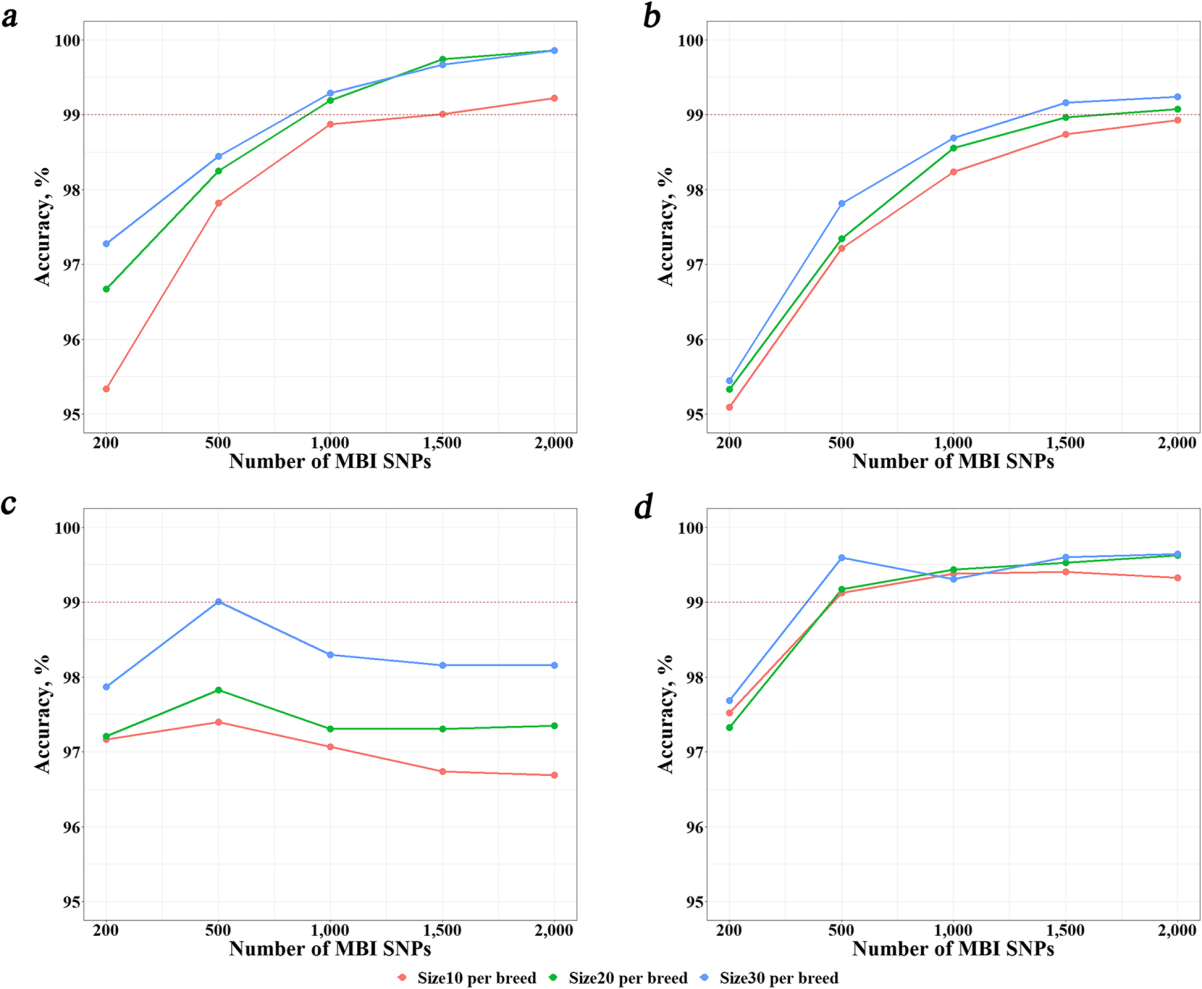
Identification accuracies with different reference population size (30, 20 and 10 individuals per breed) using the most breed-informative (MBI) SNPs revealed by DFI. **a**, **b**, **c** and **d** refer to machine learning methods KNN, RF, SVM and KSR, respectively. *KNN* K-Nearest Neighbor, *RF* Random Forest, *SVM* Support Vector Machine, *KSR* An integration of KNN, SVM and RF

The accuracies of KNN, RF, and SVM for different of reference population sizes (30, 20 and 10 bulls per breed) were shown in Fig. 3 and Additional file 1: Table S3. Here, only the MBI SNPs from the DFI method was used. In general, the accuracies increased when the reference population getting larger. However, the differences were generally small. Even for size of 10 bulls per breed, the accuracies could reach over 95% to over 99%. Since there was no method which performed the best or the worst in all situations, we tried to integrate the three method by taking the intersection of their results, i.e., intersection of all of the three methods or intersection of any two of them. If there was no intersection at all, we took the result of KNN because it performed the best in most cases. We named this method KSR. It can be seen that this method slightly increased the accuracy in almost all cases, especially in cases of reference population size of 10 bulls per breed. With KSR, the accuracies reached over 99% in all situations except the number of MBI SNPs being less than 500. Therefore, KSR was more robust than any single method.

#### Impact of number of breeds on breed identification

To explore whether the number of breeds involved in the breed identification has an impact on the accuracy of breed identification, we compared the accuracies of breed identification when the number of breeds were 3, 5, 10 and 13. The results are given in Additional file 1: Table S4. In general, the identification accuracy decreased as the number of breeds increased. The more breeds involved, the more MBI SNPs are needed to obtain high accuracy. However, it should be noted that the accuracy also depends on the breed purity of the animals in the reference and validation population.

### Both reference and test populations genotyped by SNP chips

**Fig. 4:**
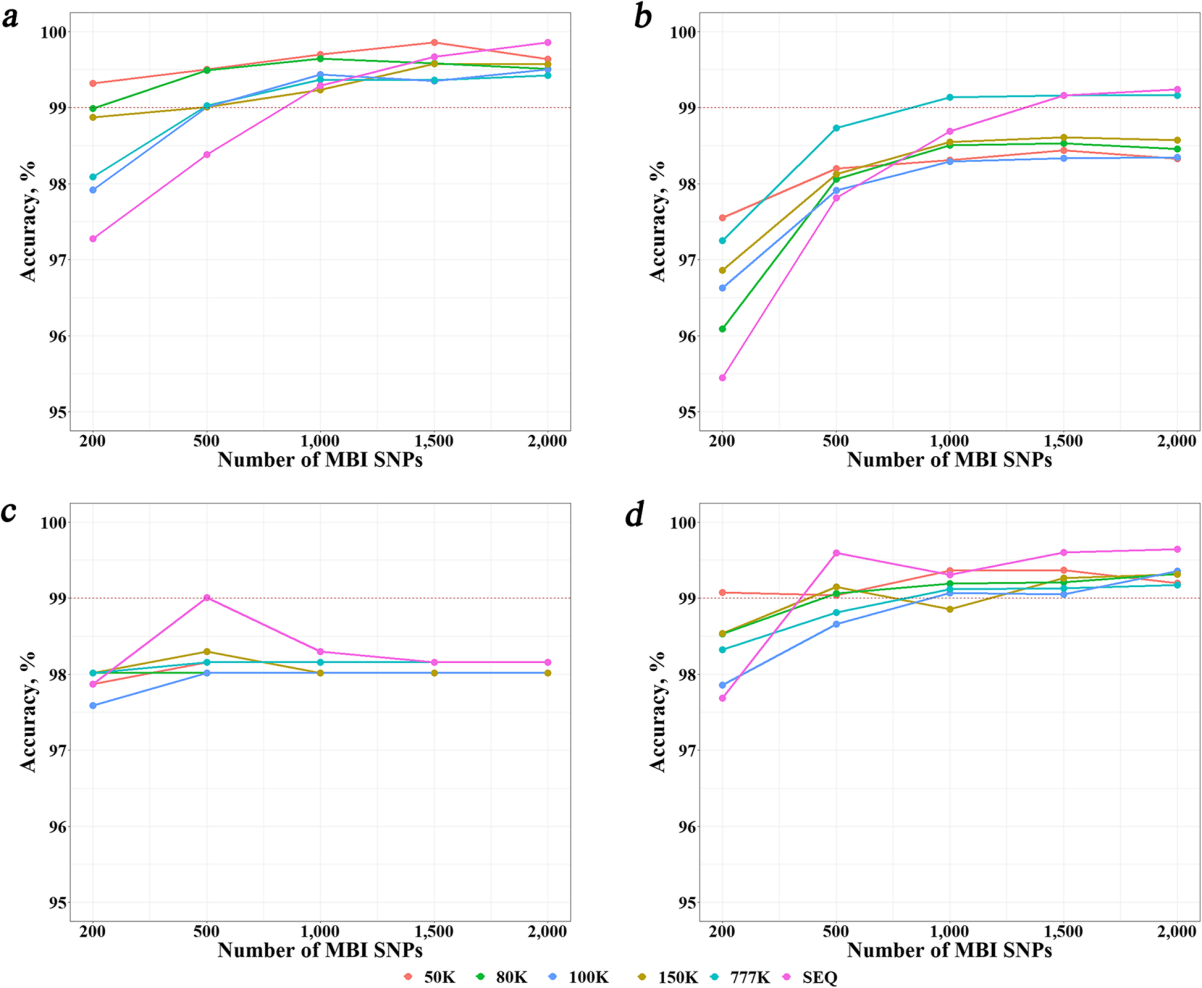
Identification accuracies with different SNP chips and sequencing data using the most breed-informative (MBI) SNPs revealed by DFI. The reference population size was 30 individuals per breed. **a**, **b**, **c** and **d** refer to machine learning methods KNN, RF, SVM and KSR, respectively. *KNN* K-Nearest Neighbor, *RF* Random Forest, *SVM* Support Vector Machine, *KSR* An integration of KNN, SVM and RF, *SEQ* Sequence data

The breed identification accuracies were assessed when both reference and test populations were genotyped with five different SNP chips (50K, 80K, 100K, 150K, and 770K). Figure 4 (and Additional file 1: Table S5) shows the results of the four machine learning methods (KNN, RF, SVM, and KSR) with the reference population of 30 bulls per breed and the MBI SNPs from the DFI method. As a comparison, the accuracies from the sequence data were also included. Several interesting observations can be drawn from the results. First, there was no clear relation between the accuracy and chip density, the accuracies using chip data were sometimes even better than that using sequence data. For KNN, the 50K and 80K chips resulted in the highest accuracies in most cases; for RF, the 777K chip performed the best in most cases; and for SVM and the integrated method KSR, the sequence data outperformed all chip data (except in case of number of MBI SNPs equal to 200). However, it should be noted that the highest accuracy among all cases was achieved by using sequence data and 2,000 MBI SNPs.

### Reference population genotyped by sequencing and test population by SNP chips

Here, the sequence data in the reference population was used to detect the MBI SNPs. For individuals in test population with chip data, we first imputed their chip genotypes to sequence level to recover their genotypes of the MBI SNPs. Machine learning classification was carried out using these imputed genotypes. Table 3 shows the identification accuracies of four machine leaning methods (KNN, RF, SVM and KSR) using 2,000 MBI SNPs from DFI. There were very small proportions (1%–13%) of the 2,000 MBI SNPs contained in the chip SNPs. The imputation accuracies for the missing MBI SNPs were 83%–94% for the five types of chip (increased with the chip densities). Although the imputation accuracies were not very high (especially for the 50K chip), the breed identification accuracies based on the imputed SNPs were comparable with that of the sequence data.

**Table 3:** Identification accuracies (Mean (SE) over 50 replications) when the reference population was genotyped with sequencing and the test population was genotyped with different SNP chips or with sequencing^a^

Chip/SEQ	No. MBI SNPs contained^b^	Imputation accuracy	Identification accuracy, %			
			**KNN**	**RF**	**SVM**	**KSR**
50K	20	83.58%	99.69 (0.01)	97.87 (0.03)	98.01 (0.00)	99.16 (0.02)
80K	52	88.52%	99.86 (0.00)	99.19 (0.04)	98.16 (0.00)	99.51 (0.03)
100K	65	89.41%	99.33 (0.01)	98.75 (0.03)	98.01 (0.00)	98.84 (0.02)
150K	91	91.16%	99.65 (0.01)	99.04 (0.03)	98.01 (0.00)	99.19 (0.03)
777K	261	94.36%	99.72 (0.00)	99.15 (0.03)	98.01 (0.00)	99.30 (0.03)
SEQ	2,000	–	99.86 (0.00)	99.24 (0.03)	98.16 (0.00)	99.65 (0.03)

^a^The chip genotypes were imputed to sequence level. The reference population size was 30 individuals per breed and 2,000 most breed-informative SNPs derived by DFI were used

^b^Number of SNPs among the 2,000 most breed-informative (MBI) SNPs derived from the reference population which were contained in the chips

*KNN* K-Nearest Neighbor, *RF* Random Forest, *SVM* Support Vector Machine, KSR, an integration of KNN, SVM and RF

### GBC estimation of the test animals

The average GBC of the test animals for the 13 breeds are given in Table 4. It can be seen that, except for the animals labelled as GEL, the average GBC of all animals were over 85% for their labelled breeds, indicating that their breed purities were high on average, especially the one animal labelled as YKT, which had almost 100% GBC for YKT. The three animals labelled as GEL had only 46.94% GBC for GEL, while they had 28.21%, 7.57% and 6.25% GBC for SIM, ANG and HF, respectively, indicating these animals were very likely crossbred animals, although they were classified as GEL. On the other hand, although the other animals had high average GBC for their corresponding labelled breeds, some of them could be also crossbred animals. As mention above, the majority of identification errors happened in BS, and the misclassified animals were all assigned to GEL*.* We checked the GBC of these misclassified animals. It turned out that they had low GBC for BS (20%–30%, Additional file 1: Table S6), which were very close to (some even lower than) their GBC for GEL.

**Table 4:** The average GBC (%) of the test animals (in rows) across the 13 breeds (in columns) estimated using 789K SNPs

**Breed**	NMD	YKT	GEL	LIM	MBL	HF	NWR	CHA	SIM	BS	JER	ANG	HOL
NMD	**92.40**	0.00	0.00	2.87	0.00	0.00	0.00	0.00	0.00	0.00	0.00	0.00	4.71
YKT	0.00	**99.99**	0.00	0.00	0.00	0.00	0.00	0.00	0.00	0.00	0.00	0.00	0.00
GEL	0.64	1.46	**46.94**	0.31	1.15	6.25	3.06	1.11	28.21	3.13	0.05	7.57	0.12
LIM	0.19	0.56	0.06	**88.57**	0.40	1.06	0.27	5.35	2.39	0.21	0.50	0.45	0.00
MBL	0.38	0.10	0.32	1.80	**92.08**	0.15	0.07	0.09	4.61	0.13	0.00	0.00	0.27
HF	0.71	0.06	0.14	0.63	0.06	**95.47**	0.53	1.09	0.41	0.00	0.06	0.76	0.07
NWR	0.86	0.18	0.49	0.27	0.04	0.46	**93.79**	0.94	0.04	0.91	0.02	0.56	1.44
CHA	0.83	0.92	0.01	2.83	0.19	3.19	0.85	**88.38**	1.09	0.47	0.42	0.78	0.05
SIM	0.39	0.33	0.50	3.81	2.89	0.58	0.47	1.40	**87.68**	0.69	0.05	0.61	0.60
BS	0.98	0.95	4.97	0.10	1.10	0.39	0.83	1.51	3.76	**84.50**	0.50	0.14	0.27
JER	0.78	0.21	0.25	1.96	0.06	0.28	0.55	0.09	0.04	0.04	**94.89**	0.07	0.78
ANG	0.45	0.10	0.17	0.21	0.02	0.87	0.88	1.04	0.02	0.01	0.04	**95.75**	0.43
HOL	1.50	0.24	2.49	0.87	0.18	0.7	2.04	0.85	0.48	0.03	0.15	0.47	**89.99**

See Table 1 for breed codes and Table 2 for number of test animals in each breed

## Discussion

In recent years, many studies have been devoted to identification of animal breeds based on SNPs. However, they focused on comparison of either different breed-informative detection methods or different machine learning classification methods [28, 42, 43]. It is valuable to explore the optimal combination of breed-informative SNPs detection strategies and machine leaning methods for breed identification. In this study, we compared three different breed-informative detection methods (Delta, Wright’s *F*_ST_ and *I*_n_) and five machine learning classification methods (KNN, SVM, RF, NB and ANN) and their combinations (pipelines). We evaluated their performance with varying reference population size and varying number of MBI SNPs. In addition, we proposed to integrate the three informative SNP detection methods by using MBI SNPs which were the common SNPs among the MBI SNPs revealed by the three methods. We found the integrated method, called DFI, performed better than or equally well as the three methods in all combinations with the machine learning methods. We also proposed to integrate the three machine learning methods, KNN, SVM, and RF, which were obviously better than the other two methods, by taking the intersection of the identification results of the three methods. This integrated method, called KSR, outperformed any of the single method in most cases and, more importantly, it was very robust with identification accuracies over 99% in all scenarios except when the number of MBI SNPs was less than 500.

In general, the identification accuracy increased with the increases of the reference population size and the number of MBI SNPs. However, for the SVM based pipelines, the highest accuracy was achieved when the number of MBI SNPs was 500 and was getting down when the number of MBI SNPs increased (Figs. 2, 3 and 4). We looked at the detailed identification errors in individual breeds and found that majority of the errors occurred in Brown Swiss (Table 2). This occurred not only for SVM, but also for all other methods except for KNN which did not make any error. The average error rate for Brown Swiss across all methods was 7.82%, while the overall average error rate across all breeds was 1.55%. Further, the misclassified Brown Swiss animals were all assigned to Gelbvieh. We computed the genetic distances among the 13 breeds using the *F*_ST_ statistic (See Additional file 1: Table S7). Brown Swiss had the closest distance with Gelbvieh and Simmental (*F*_ST_ = 0.13). The GBC analysis for the misclassified BS animals showed that they had low GBC (20%–30%) for Brown Swiss, which were close to (some even lower than) their GBC for Gelbvieh. This led to the miss classification of these animals to Gelbvieh.

In farm animal society, different types of SNP chips have been widely used for genome genetic analysis, which produced abundant genome data available. These data were also used for breed identification [16, 44–46]. To compare the accuracies using sequence data and chip data in breed identification, we generated cattle chip data of five different densities (from 50 to 777K). It turned out that for the KNN method, it was the 50K chip which produced the highest accuracies in most cases, while for the RF and SVM methods, it was the sequence data which produced the highest accuracies in most cases. There was no clear relation between chip densities and accuracies. However, for all methods and SNP chip types, the accuracies could reach over 97% except for the RF method and the number of MBI SNPs was less than 500. Therefore, SNP chips are also good options for breed identification. This is consistent with the conclusions of previous studies [12, 28, 45], in which high accuracies (generally over 95%) of breed identification were obtained by using SNP chip data. In addition, we also evaluated the situation where the reference population was genotyped with sequencing and the test population was genotyped with SNP chips, the results showed that, by imputation of the chip data to sequence data, almost the same accuracies could be obtained as the situation where both reference and test population were genotyped with sequencing.

It would be interesting to know whether there are some pathways involved in the breed diversification. We performed Kyoto Encyclopedia of Gene and Genome (KEGG) pathway analysis for genes in the vicinity of the 1,000 MBI SNPs using Database for Annotation, Visualization and Integrated Discovery (DAVID) [47]. Five hundred and eighty-one genes were involved in this analysis, and 9 significant pathways (*P* < 0.05) were identified (Additional file 1: Table S8). Some pathways could be involved in breed diversification. For example, the Melanogenesis pathway and the NF-kappa B signaling pathway could be related to hair color and stress resistance, respectively, which are regarded as important characteristics of a breed. However, it is hard to find general clear relationship between these pathways and breed characteristics, although there are some SNPs showing strongly associated with some breed characteristics. For example, the SNP in the *KIT* gene in the Melanogenesis pathway, which has been proved as a functional gene for hair color, had high frequency (0.7–1.0) for allele C in breeds with white pieces, like HOL, NMD, MBL, and HF, while it had frequency of zero (or nearly zero) in breeds without white pieces, like ANG, LIM, GEL, and BS.

## Conclusions

We compared different combinations of breed-informative SNPs detection methods (Delta, *F*_ST_, and *I*_n_) and machine leaning classification methods (KNN, RF, SVM, NB, and ANN) for breed identification using sequence and SNP chip data with respect to different reference population sizes and number of most breed-informative SNPs. We found that, although in all scenarios the identification accuracies could reach over 95%, the combination of DFI (an integration of Delta, *F*_ST_, and *I*_n_) and KSR (an integration of KNN, SVM, and RF) was the optimal strategy, which produced the highest accuracies in most cases (over 99%) and was very robust across all scenarios. Generally, the accuracies increased along with the increase of the reference population size and the number of most breed-informative SNPs. Using sequence data resulted in higher accuracies than using chip data in most cases. However, the differences were generally small. In view of the cost of genotyping, using chip data is also a good option for breed identification.

## Supplementary Information

**Additional file 1:Table S1.** Summary of the machine learning classification models. **Table S2.** Accuracies of different pipelines for breed identification with different most breed-informative SNPs. **Table S3.** Accuracies of difference machine learning methods with respect to different reference population size and different numbers of most breed-informativeSNPs revealed by DFI. **Table S4.** Accuracies of difference machine learning methods with respect to different number of breeds. The reference population size was 30 individuals per breed and the most breed-informativeSNPs were from DFI. **Table S5.** Accuracies when both reference and test population were genotyped with SNP chipsand with sequence. The reference population size was 30individuals per breed and the most breed-informativeSNPs were from DFI. **Table S6.** The GBCof the misclassified Brown Swiss animals estimated using 789K SNPs. **Table S7.** Distance matrixamong the 13 cattle breeds. **Table S8.** Pathway enrichment of genes in the vicinity of the 1,000 most breed-informative SNPs.

## Data Availability

All data supporting our findings are included in the manuscript.

## Abbreviations

ANN: Artificial Neural Network
DAVID: Database for Annotation, Visualization and Integrated Discovery
DFI: An integration of Delta, *F*_ST_, and *I*_n_
GBC: Genomic breed composition
KEGG: Kyoto Encyclopedia of Gene and Genome
KNN: K-Nearest Neighbor
KSR: An integration of KNN, SVM, and RF
LD: Linkage disequilibrium
MAF: Minor allele frequency
MBI: Most breed-informative
NB: Naïve Bayes
RF: Random Forest
SVM: Support Vector Machine
